# Connexin Expression Is Altered in the Eye Development of *Yotari* Mice: A Preliminary Study

**DOI:** 10.3390/biom14091174

**Published:** 2024-09-19

**Authors:** Ljubica Skelin, Anita Racetin, Nela Kelam, Marin Ogorevc, Ljubo Znaor, Mirna Saraga-Babić, Natalija Filipović, Yu Katsuyama, Zenon Pogorelić, Katarina Vukojević

**Affiliations:** 1 Clinical Department of Ophthalmology, University Hospital of Split, 21000 Split, Croatia; lskelin@kbsplit.hr (L.S.);; 2 Department of Anatomy, Histology and Embryology, University of Split School of Medicine, Šoltanska 2A, 21000 Split, Croatiakatarina.vukojevic@mefst.hr (K.V.); 3 Center for Translational Research in Biomedicine, University of Split School of Medicine, Šoltanska 2A, 21000 Split, Croatia; 4 Department of Ophthalmology, University of Split School of Medicine, Šoltanska 2A, 21000 Split, Croatia; 5 Department of Anatomy, Shiga University of Medical Science, Otsu 520-2192, Japan; 6 Department of Pediatric Surgery, Split University Hospital, 21000 Split, Croatia

**Keywords:** *yotari*, developing eyes, retinogenesis, connexins

## Abstract

This study aimed to explore how *Dab1* functional silencing influences the expression patterns of different connexins in the developing *yotari* (*yot*) mice eyes as potential determinants of retinogenesis. Using immunofluorescence staining, the protein expression of Dab1, Reelin, and connexin 37, 40, 43, and 45 (Cx37, Cx40, Cx43, and Cx45) in the wild-type (wt) and *yot* eyes at embryonic days 13.5 and 15.5 (E13.5 and E15.5) were analyzed. Different expression patterns of Cx37 were seen between the wt and *yot* groups. The highest fluorescence intensity of Cx37 was observed in the *yot* animals at E15.5. Cx40 had higher expression at the E13.5 when differentiation of retinal layers was still beginning, whereas it decreased at the E15.5 when differentiation was at the advanced stage. Higher expression of Cx43 was found in the *yot* group at both time points. Cx45 was predominantly expressed at E13.5 in both groups. Our results reveal the altered expression of connexins during retinogenesis in *yot* mice and their potential involvement in retinal pathology, where they might serve as prospective therapeutic targets.

## 1. Introduction

Eye development in mice begins at embryonic day 8 (E8) with the formation of bilateral optic sulci (pits) in the prospective forebrain [1] and continues until the eye stops growing at postnatal day 40 [2]. The retina originates from the neuroectoderm, and the choroid arises from the surrounding mesenchyme [3,4,5]. Optic nerves extend from the op-tic disc to the optic chiasm and contain glial cells and retinal ganglion cells (RGCs) axons [6]. RGCs are able to collect visual information and transmit it to the visual centers in the brain for its further processing [6]. Eye development involves a large number of interdependent processes, different gene expressions, and membrane cell signalization [4,5,7,8]. Prenatal migration of neurons in the brain is influenced by the Reelin signaling pathway acting on intracellular adaptor protein Disabled-1 (Dab1) that stimulates essential processes for developmental cell activities. *Yotari* (*yot*) is an autosomal recessive mutant of the *Dab1* gene in mice with a phenotype very similar to Reeler mice, characterized by tremors, unstable gait, central nervous system impairment, and premature death [9,10]. Considering that the retina forms as an extension of the forebrain, disruption of the Reelin–Dab1 signaling pathway may cause disturbances in retinogenesis [11].

Transmembrane proteins called connexins form junctions between two neighboring cells and are necessary to maintain metabolic homeostasis [8,12]. Recent observations suggest that gap junctions contribute to progressive cell death as well as irregular activity in distinct pathologies of the retina [12]. In the case of diabetic retinopathy, previous reports indicate that disruption of connexin-mediated cellular communication plays a crucial role [13]. Connexin 37 (Cx37) is expressed in vascular endothelial cells, creating intercellular channels among them, and is necessary for normal differentiation and early retinal angiogenesis [8,13]. Like Cx37, Cx40 forms intercellular junctions between endothelial cells, which is important for motor tone in vessels and electric heart conduction [13]. Deletion of Cx40 disrupts retinal vessel development and reduces the density of the capillaries in the neovascularized neonatal mouse retina [8,13]. Cx43 was found in retinal endothelial cells, pericytes, smooth muscle cells, retinal pigment epithelium (RPE), microglia, Müller cells, and astrocytes. The role of Cx43 is known in glaucoma, diabetic retinopathy, and macular degeneration [14]. Recent studies have shown that Cx43 hemichannels can mediate RPE disruption and, consequently, the blood–retina barrier integrity issue that occurs in diabetic macular edema [14]. During development, Cx45 is expressed in bipolar cells, where it is involved in heterologous electrical synapses composed of some retinal ganglion cells, which need intercellular communication for their complex and precisely coordinated functions [15,16].

Due to its neuroectodermal origin and the abundance of gap junctions in the mouse retina, we were interested in connexin expression during retinogenesis in the *yot* mouse compared to the wild type (wt). Understanding the importance of gap junctions and their constituent parts as well as connexins in the tissue of the complexly structured retina can lead to the development of potential novel therapeutical targets.

## 2. Materials and Methods

### 2.1. Ethics

The experiment was approved by the Guidelines for the Care and Use of Laboratory Animals at the Shiga University of Medical Science. The study was conducted according to the guidelines of the Declaration of Helsinki and approved by the Ethical Committee of the University of Split School of Medicine (UP/1-322-01/17-01/13; 525-10/0255-17-7).

### 2.2. Animal Handling and Sample Preparation

*Yotari* mice are Dab1-null conventional mutants previously described by Sheldon et al. [10]. PCR primers used for genotyping the mice were *yotari*: GCCCTTCAGCATCAC-CATGCT and CAGTGAGTACATATTGTGTGAGTTCC; the wild type of *Dab1* locus: GCCCTTCAGCATCACCATGCT and CCTTGTTTCTTTGCTTTAA-GGCTGT [17]. C57BL/6 N mice were housed in standard polycarbonate cages with ad libitum access to food and water in a temperature-controlled (23 ± 2 °C) room with a 12 h light/dark cycle. The gravid mice were anesthetized with pentobarbital and transcardially perfused using phosphate buffer saline (PBS, pH 7.2) and 4% paraformaldehyde (PFA) in 0.1 M PBS. Embryos were collected at 13.5 and 15.5 gestation days (E13.5 and E15.5), fixed in 4% PFA in 0.1 M PBS overnight for conventional histological analyses: hematoxylin–eosin (H&E) and immunofluorescence (IF) staining [18,19]. In summary, we used thirteen animals: three each in the E13.5 wt, E13.5 *yot*, and E15.5 *yot* and four in E15.5 wt).

### 2.3. Immunofluorescence Staining

The paraffin-embedded tissue was sliced in 5 µm thick consecutive sections, and every 10th section was stained with H&E staining to verify adequate tissue preservation. After deparaffinization and rehydration, antigen retrieval was performed in a water steamer with sodium citrate buffer (pH 6.0) for 20 min. For the nonspecific binding prevention, a protein blocking buffer (ab 64226, Abcam, Cambridge, UK) was applied for 30 min at room temperature, after which samples were covered with primary antibodies (Table 1) in a humidity chamber overnight. The following day, suitable secondary antibodies (Table 1) were applied for one hour at room temperature and protected from the light. Slides were stained with 4,6-diamidino-2-phenylindole (DAPI) for nuclei detection and cover-slipped (Immuno-Mount, Thermo Scientific, Thermo Shandon, Cheshire, UK). No staining was observed when primary antibodies were excluded from the experimental procedure.

### 2.4. Data Acquisition and Statistical Analysis

The analysis was performed with an epifluorescence microscope (Olympus BX51, Tokyo, Japan) equipped with a Nikon DS-Ri2 camera (Nikon Corporation, Tokyo, Japan). Ten representative visual fields of the mouse embryonic eye per group were captured with the same camera settings using 40× magnification. Only the retina was included in the analysis. By using the Lasso tool in Adobe Photoshop (Adobe, San Jose, CA, USA) in all images, the retina was separated from the surrounding tissue. ImageJ software, version 1.54 (NIH, Bethesda, MD, USA), was used to process images to isolate the positive signal. The resulting images were thresholded using the “triangle” method. The area percentage of the thresholded images was determined using the “analyze particles” function. Significant parts of all analyzed images were devoid of any tissue, and a correction of the area percentage was necessary to calculate the actual area percentage as it is described previously [20].

Fluorescence intensity histograms were acquired for the green fluorescence channel. Expression of different markers was quantified as the area under the curve (AUC) of fluorescence intensity histograms. The threshold for background exclusion was set on 30 fluorescence intensity units. AUC data were normalized on the median of the E13.5 wt group for each of the observed proteins.

Statistical analyses were performed using GraphPad Software 8.0 software (GraphPad Software, La Jolla, CA, USA) with the probability level of *p* < 0.05 considered statistically significant. The Shapiro–Wilk test was used to check normal distribution. A two-way ANOVA test followed by post hoc Tukey’s test was performed to compare the expression of the observed proteins between groups. Kruskal–Wallis test followed by Dunn’s multiple comparison test was performed to compare AUC values between groups. *p* < 0.05 was considered statistically significant.

## 3. Results

### 3.1. Morphological Characteristics of Yotari Mice Eye and Optic Nerve Development

**Figure 1:**
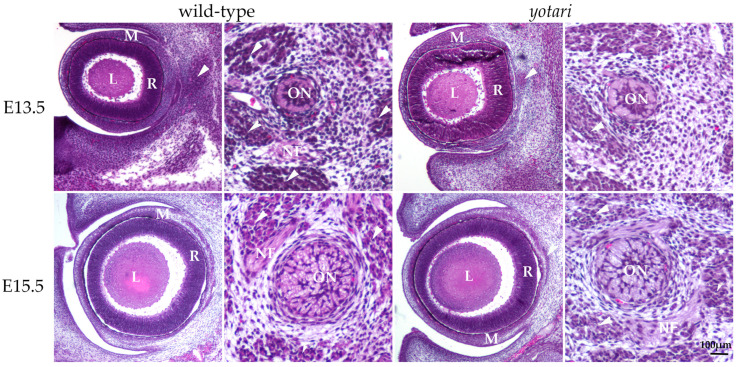
Morphology of developing wild-type (wt) and *yotari* (*yot*) eyes and optic nerve. The mesenchyme will give rise to the choroid and sclera. There are no significant morphological differences between wt and *yot* eyes. L—lens, M—mesenchyme, R—retina, arrows—extraocular muscles, ON—developing optic nerve, NF—nerve fibers, and arrows—developing extraocular muscles. Scale bar is 100 μm, which refers to all images.

The E13.5 wt eye contained all characteristic layers, including the neural and pigmented retina, which differentiated from the optic cup, as well as the lens derived from the lens vesicle. The surrounding mesenchyme gradually differentiated into the choroid coat of the eyeball (choroid, ciliary body, and iris) and the fibrous coat, consisting of the sclera and cornea. The blood vessels and external muscles differentiated from the mesenchyme as well. The nuclei of differentiating retinal cells appeared homogenous and without stratification (Figure 1). In the *yotari* mice, the same parts of the eye were observed, without any significant morphological differences when compared to the wt group (Figure 1).

In the 13.5 wt mice, the optic nerve, differentiating from the optic stalk, consisted of unmyelinated axons of ganglion cells and a small central lumen surrounded by nuclei of immature glial cells. In the surrounding mesenchyme, differentiating muscles, blood vessels, and leptomeningeal layers were observed (Figure 1). A similar appearance of optic nerve morphology was observed in the *yot* mice of the same developmental stage (Figure 1).

At E15.5, differentiation advancement was observed in the structures comprising the eyeball of wt mice. In the neural retina, differentiation of ganglion cells was observed at the luminal surface, facing the vitreous compartment of the eye, while other layers still appeared homogenous. Mesenchymal derivatives such as muscles, the choroid, and the connective tissue parts of the cornea and sclera were distinguishable. The differentiation of nuclear lens fibers was also observed (Figure 1). In *yot* mice, the same ocular structures were observable without significant morphological differences compared to the wt group (Figure 1).

In the 15.5 wt mice, advancement in the optic nerve diameter was associated with loss of the central lumen and an increase in axons, which appeared bubbly. Axons were separated with loose connective tissue of the pia mater. Further, an increase in concentric organization of meninges was observed around the optic nerve (Figure 1). In *yot* mice of the same stage, advancement in optic nerve differentiation appeared less extensive than in normal development, while the nerve fibers appeared less bubbly (Figure 1).

### 3.2. Dab1 and Reelin Expression in the Eye of Wild-Type Mice

**Figure 2:**
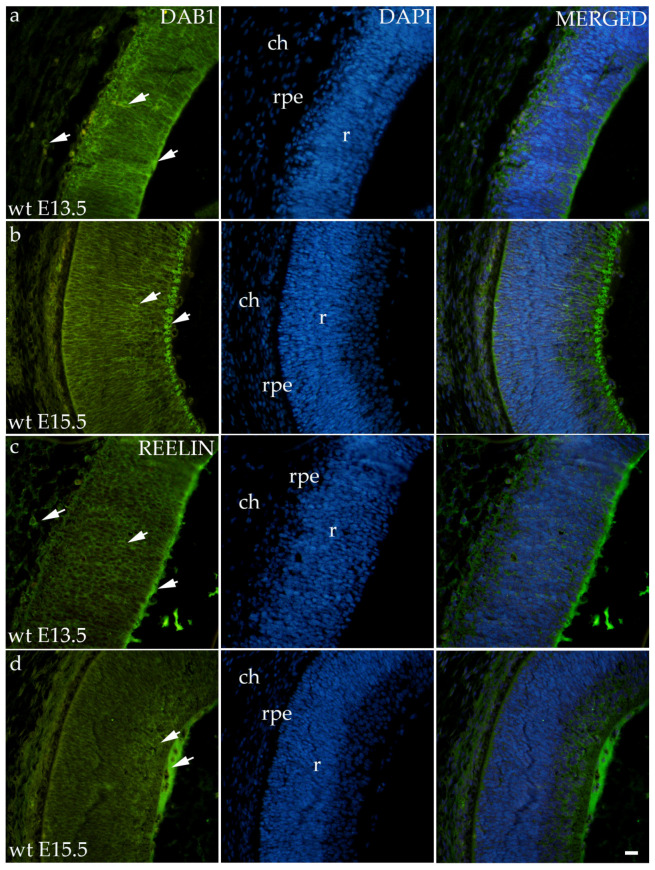
Immunohistochemical expression patterns of Dab1 and Reelin markers in the eyes of wild-type (wt) mice at embryonic day 13.5 (E13.5) (**a**,**c**) and embryonic day 15.5 (E15.5) (**b**,**d**). Arrows show positive staining of the cells through the eyeball layers. r—retina, rpe—retinal pigment epithelium, and ch—choroid. The scale bar is 20 μm, which refers to all images.

Dab1 and Reelin have similar expression patterns in wild-type animals in both time points (Figure 2). At E13.5, Dab1 and Reelin were expressed through all the layers of the developing retina including RPE and the choroid. At the E15.5, staining was predominantly located at the apical layer of the retina, i.e., the future neural fiber layers (Figure 2).

### 3.3. Expression Patterns of Different Connexins

#### 3.3.1. Connexin 37

**Figure 3:**
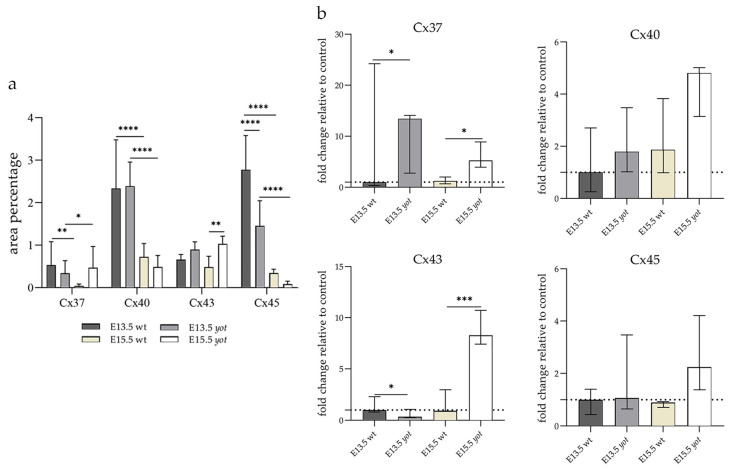
The area percentages of different connexins in the retina of wild-type (wt) and *yotari (yot)* mice at embryonic day E13.5 and E15.5 (**a**). Fluorescence intensity histograms comparing expression of different connexins in the retina of wild-type and *yotari* mice at embryonic day E13.5 and E15.5 (**b**). Data are presented as the mean ± SD (vertical line) (**a**) and median with interquartile range (**b**). For the baseline, we used values calculated for E13.5 wt. Significant differences were indicated by * *p* < 0.05, ** *p* < 0.01, *** *p* < 0.001, **** *p* < 0.0001. One-way ANOVA followed by Tukey’s multiple comparisons test or Kruskal–Wallis followed by Dunn’s multiple comparisons test were performed.

The area percentage of Cx37 was deficient within all observed groups, with the highest count in the E13.5 wt animals. Statistical significance was observed in wt animals between E13.5 and E15.5 as well as in the *yot* group (Figure 3a). Higher AUC values were observed in the *yot* groups of both observed time points (*p* < 0.05, Figure 3b).

**Figure 4:**
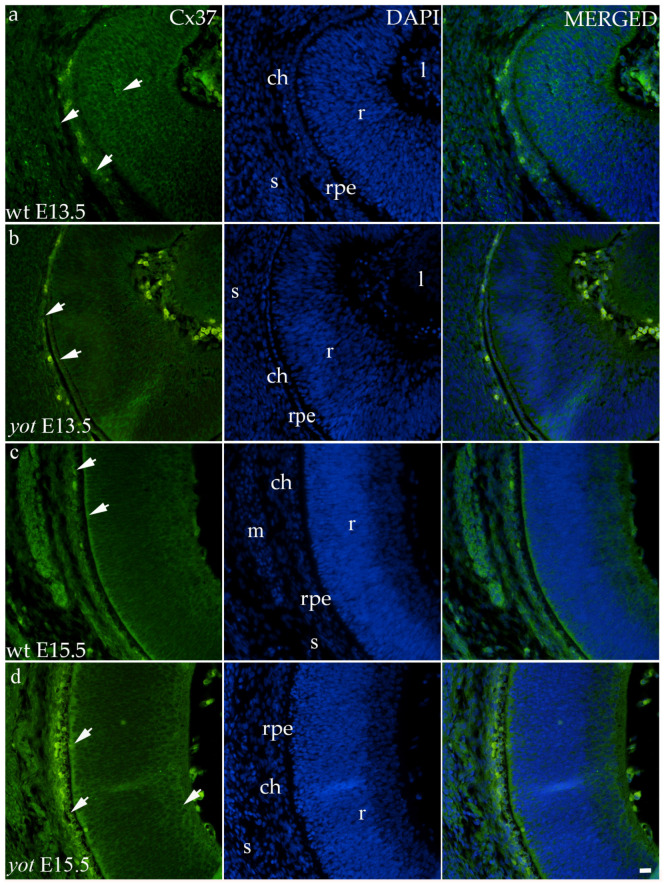
Immunohistochemical expression patterns of Cx37 marker in the eyes of wild-type (wt) and *yotari* (*yot*) mice group at embryonic day 13.5 (E13.5) (**a**,**b**) and embryonic day 15.5 (E15.5) (**c**,**d**). Arrows show positive staining of the cells through the eyeball layers. r—retina, rpe—retinal pigment epithelium, ch—choroid, s—sclera, and m—muscles, l—lens. The scale bar is 20 μm, which refers to all images.

Regarding the distribution of Cx37, different spatial patterns were seen between the wt and *yot* groups (Figure 4). In the E13.5 wt group, mild expression was seen in the middle layers of the neural retina, choroid, and sclera (Figure 4a), whereas in the E13.5 *yot* group, most of the expression was localized in the RPE and choroid (Figure 4b). At E15.5, most of the expression in both experimental groups was seen in the basal part of the neural retina, choroid (Figure 4c,d), and muscles (Figure 4c).

#### 3.3.2. Connexin 40

**Figure 5:**
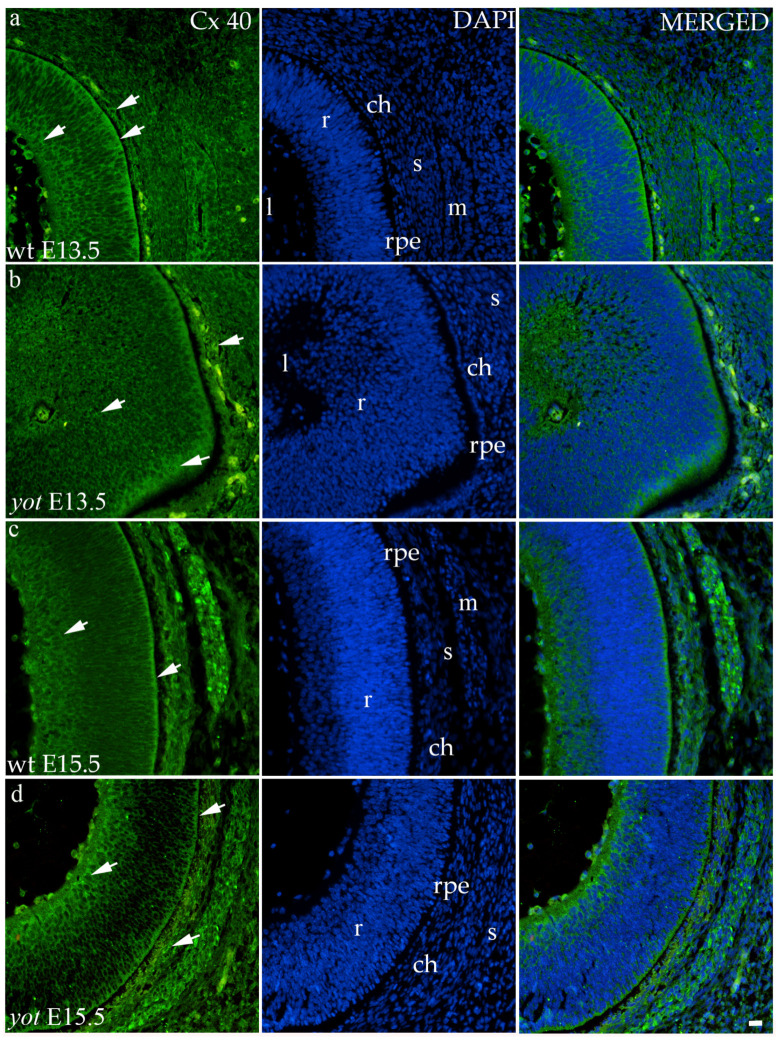
Immunohistochemical expression patterns of Cx40 marker in the eyes of wild-type (wt) and *yotari* (*yot*) mice group at embryonic day 13.5 (E13.5) (**a**,**b**) and embryonic day 15.5 (E15.5) (**c**,**d**). Arrows show positive staining of the cells through the eyeball layers. r—retina, rpe—retinal pigment epithelium, ch—choroid, s—sclera, and m—muscles, l—lens. The scale bar is 20 μm, which refers to all images.

The highest area percentage of Cx40 was in the E13.5 *yot* group, with statistical significance in comparison to the other groups (*p* < 0.05, Figure 3a). Generally, in both groups, higher expression of this protein was observed at the E13.5 during early differentiation of retinal layers, whereas it decreased at E15.5 (*p* < 0.05, Figure 3a), when differentiation was at an advanced stage. There was no statistically significant difference in AUC between wt and *yot* animals at any observed time point (*p* < 0.05, Figure 3b). Also, the spatial expression pattern of Cx40 was quite coherent in all experimental animal groups (Figure 5). Most of the expression was localized in the retinal apical and basal layers, choroid, and muscles (Figure 5a–d). Interestingly, although there was no statistically significant difference in fluorescence intensity overall, the intensity of Cx40 expression seemed to be higher in the basal region of the retina of the wt group compared to the *yot* group at E13.5 (Figure 5a,b). At E15.5, however, the apical retinal layer of the *yot* group displayed stronger intensity than the wt group (Figure 5c,d).

#### 3.3.3. Connexin 43

**Figure 6:**
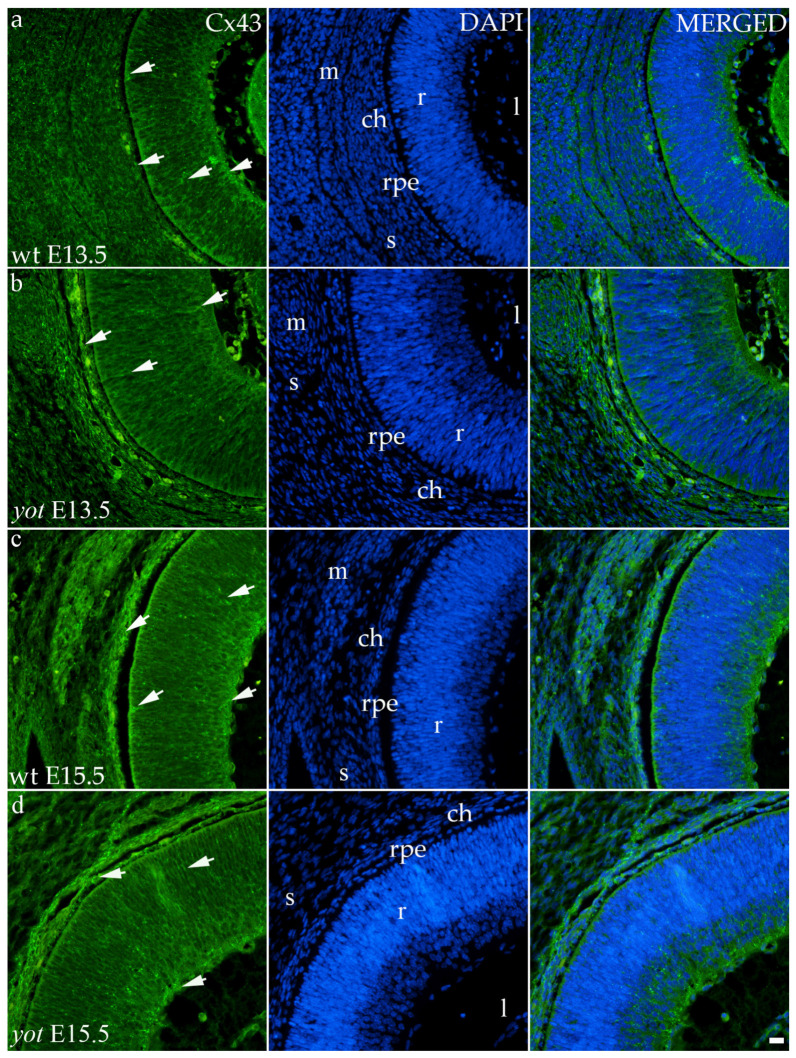
Immunohistochemical expression patterns of Cx43 marker in the eyes of wild-type (wt) and *yotari* (*yot*) mice group at embryonic day 13.5 (E13.5) (**a**,**b**) and embryonic day 15.5 (E15.5) (**c**,**d**). Arrows show positive staining of the cells through the eyeball layers. r—retina, rpe—retinal pigment epithelium, ch—choroid, s—sclera, and m—muscles, l—lens. The scale bar is 20 μm, which refers to all images.

Although the area percentage of Cx43 and AUC data were quite uniform in all observed groups, the highest values were in the E15.5 *yot* group, with statistically significant difference in comparison with the E15.5 wt group (*p* < 0.05, Figure 3a,b). Concerning the distribution of expression, Cx43 in wt animals at both time points was mainly represented in the choroid, basal, and apical layers of the neural retina and occasionally in the cells of middle layers (Figure 6a,c). While the spatial expression pattern in *yot* mice was similar, there was no observable signal in the basal part of the neural retina (Figure 6b,d).

#### 3.3.4. Connexin 45

Cx45 was predominantly expressed in the E13.5 wt group compared to the other groups (*p* < 0.05, Figure 3a). The retina of the *yot* mice have higher area percentage at E13.5 than E15.5 (Figure 3a). Regarding the AUC, there was no statistically significant difference between wt and *yot* animals at any observed time points (*p* < 0.05, Figure 3b).

**Figure 7:**
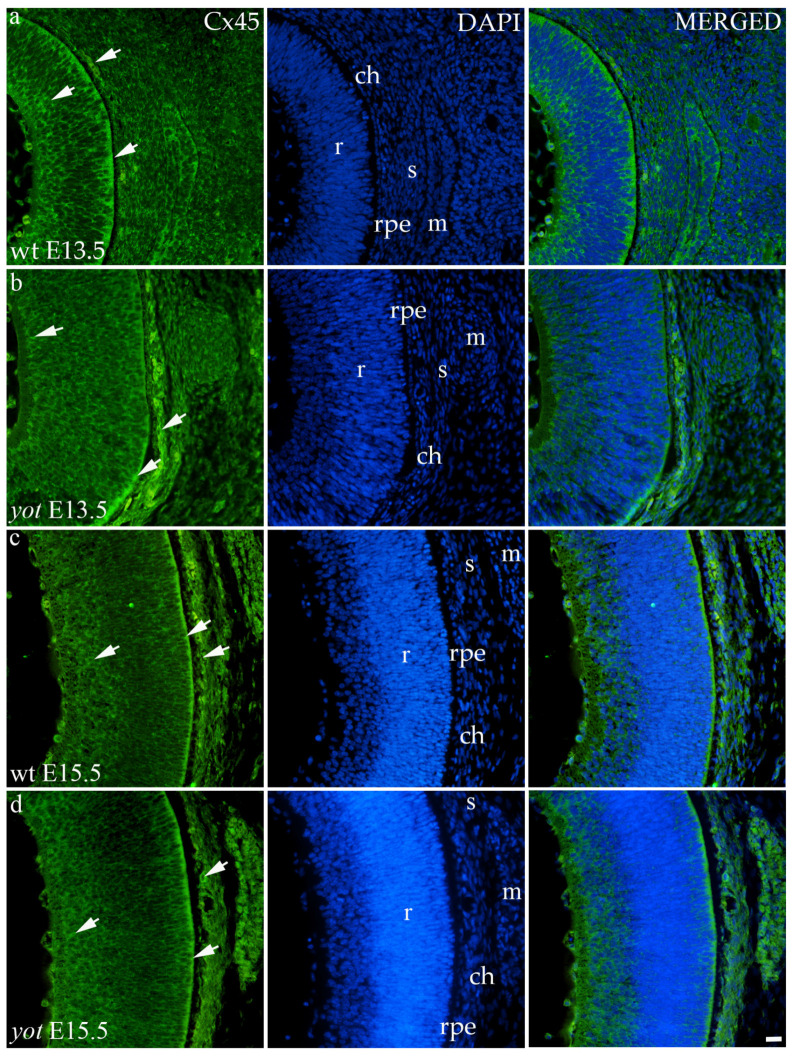
Immunohistochemical expression patterns of Cx45 marker in the eyes of wild-type (wt) and *yotari* (*yot*) mice group at embryonic day 13.5 (E13.5) (**a**,**b**) and embryonic day 15.5 (E15.5) (**c**,**d**). Arrows show positive staining of the cells through the eyeball layers. r—retina, rpe—retinal pigment epithelium, ch—choroid, s—sclera, and m—muscles. The scale bar is 20 μm, which refers to all images.

Concerning the localization of Cx45, in all analyzed groups, it was mainly distributed in the choroid, basal, and apical layers of the neural retina and muscles (Figure 7a–d). The expression of Cx45 in the retina of E13.5 wt embryos was much stronger compared to the other analyzed groups. When comparing Cx45 expression in the choroid, *yot* embryos demonstrated a seemingly stronger expression at E13.5 than wt embryos (Figure 7a,b), while there was no apparent difference between E15.5 embryos (Figure 7c,d).

## 4. Discussion

The Reelin—Dab1 pathway has been mainly investigated in the field of brain development [21,22,23,24,25]. Considering that the neurosensory retina is formed as an extension of the forebrain, disruption of the Reelin—Dab1 signaling pathway may also cause disturbances in retinogenesis [11].

In our study, Dab1 and Reelin are highly expressed through all layers of the retina and choroid and have similar expression pattern with the connexins. These results are following previous findings that have shown that, during retinogenesis, Reelin is highly expressed and responsible for the positioning and differentiation of retinal cells. In adulthood, its expression only increases after injuries and during degenerative processes [26,27].

Previous structural analysis of the retina in the Dab1-deficient mice showed a reduced density of amacrine dendrites and modifications in the layering of the amacrine cells in the area of the inner plexiform layer as well as a decrease in density of rod bipolar cells (RBC), which transduces a signal from rods to the retinal ganglion cells (RGC), influencing the assumption that RBC deficiency in *yot* mice may affect the normal activity of RGC [21]. Despite the aforementioned studies and the fact that developing retinal ganglion cells express Reelin, which is important for retinogenesis and tissue remodeling in the retina [28], at the observed time points of E13.5 and E15.5, there were no observable morphological differences in the developmental retina between *yot* and wt mice.

Similar to the brain, the neurons of the developing retina are arranged in highly organized distinct layers that will develop into a stratified appearance in which gap junctions play an important role [29]. Electrical synaptic transmission is the main mode of intercellular communication in the retina, where neurons are rich in gap junctions expressing various connexins [30]. Therefore, we wanted to examine if there is a connection between connexins and the Reelin–Dab1 signaling pathway in the retina of developing mice, especially because our previous results showed a disturbance of Cx expression in *yot* mice [31,32]. Recent observations have suggested that channels formed by connexins to enable communication between cells and the microenvironment may have an impact on the regulation of vascular permeability in the retina, a major factor in some retinal diseases [33]. Also, it was shown that the maintenance of nerve progenitor cells in a state of proliferation depends on gap junctions containing connexins [34,35].

In our analysis, we investigated both the surface distribution of the signal within the retinal tissue and the intensity of the signal itself, which we present as fold change. The higher expression of Cx37 was observed at the E13.5 in both groups, which could be caused by the beginning of the preparation of the retina for vascularization. Expression of Cx37 in the *yot* mice remained high at E15.5, and it may potentially lead to disturbed early retinal angiogenesis, in line with the results of Hamard et al., which showed the growth of aberrant retinal neovascularization and abnormalities of pericytes and smooth muscle cells if Cx37 function is impaired [13]. Examining vascularization factors during *yot* eye development would be necessary to prove this assumption. Also, different spatial expression patterns of Cx37 can be seen depending on the developmental stage, but this was al-ready observed in the earlier studies in the developing human eye [8].

The expression of Cx40 and Cx37 in blood vessels is co-regulated, and a significant balance between them is necessary to initiate proper vascular development. Their role in angiogenesis is the opposite since Cx40 stimulates and Cx37 slows down the formation of capillaries and vice versa; Cx37 stimulates and Cx40 slows down the maturation by recruiting smooth muscle cells and pericytes [13]. Similar results were obtained in our study. Namely, higher expression of Cx40 positive cells was observed during the early differentiation of retinal layers at the E13.5, in contrast to the advanced stage of differentiation at the E15.5 embryonic days. To conclude, Cx37 and Cx40 can take part in the pathogenesis of diabetic retinopathy, the first cause of blindness in developed countries [33], and a more comprehensive investigation of *yot* mice eye development should be performed due to the reduced expression of aforementioned Cx in *yot* group.

In our study, the highest expression of Cx43 was observed in the E15.5 *yot* group. In-creased opening of Cx43 hemichannels, followed by the initiation of an inflammatory response, is usually associated with diabetic retinopathy and age-related macular degeneration [36]. Considering that the expression of Cx43 had a subtle elevation in the *yot* group at E15.5, we assume there could be slight inflammation in the retina of the *yot* mice, but additional experiments are necessary to prove this assumption. Also, the same distribution pattern of Cx43 was found in wt and *yot* mice, except that there was no visible signal in the basal part of the neural retina in *yot* mice. These results could be a good basis for further research on the development and functionality of the *yot* eye because a previous study showed that lack of Cx43 causes developmental issues in retinal progenitor cells and, consequently, retinal defects [37]. Such developmental problems lead to oculodentodigital dysplasia, microphthalmos, glaucoma, strabismus, and blindness [37].

In our investigation, Cx45 was significantly expressed in the E13.5 wt group compared to the other groups. Also, it was mainly distributed in the apical and basal layers of the neuroretina, choroid, and muscles in all analyzed groups. This finding is consistent with our previous study, in which Cx45 showed the highest expression during early eye development in the human retina and decreased later [8]. When comparing Cx45 expression in the choroid, *yot* mice demonstrated stronger expression at E13.5 than wt embryos, while there was no apparent difference between E15.5 groups. It was already shown that astrocytes and Muller cells can express Cx45, but most of it can be expressed by developing neurons [38]. Also, mice amacrine and horizontal cells express Cx45 [39,40]. Because of this broad expression of Cx45 in different cellular types, a group of authors suggested a possible physiological role of Cx45 in apoptosis and calcium wave activity, which plays an important part in the development of amacrine and ganglion cells [39].

The main limitation of our study is its observational nature. Due to the fact that our samples were paraffin-embedded and formalin-fixed, we could not perform procedures for quantitative expression analysis, such as qPCR, flow cytometry, or Western blotting. Although we currently analyzed the expression of Cx37, 40, 43, and 45 at E13.5 and E15.5, examining the expression of the observed proteins in the later developmental phases would surely provide significant benefits.

## 5. Conclusions

In summary, our investigation found no discernible morphological alterations in the eyes of *yot* mice at embryonic stages E13.5 and E15.5. However, notable differences in the expression patterns of the examined connexins were evident, suggesting potential involvement in specialized roles in intercellular communication, functional integrity, and the preservation of retinal homeostasis.

## Figures and Tables

**Table 1:** Primary and secondary antibodies used in the study.

	Antibodies	Host	Dilution	Source
Primary	Anti-Cx37/GJA4	Rabbit	1:300	ab181701; Abcam, Cambridge, UK
	Anti-Cx40/GJA5	Rabbit	1:50	ab213688; Abcam, Cambridge, UK
	Anti-Cx43/GJA1	Goat	1:100	ab87645; Abcam, Cambridge, UK
	Anti-Cx45/GJA7	Rabbit	1:500	ab135474; Abcam, Cambridge, UK
	Reelin (E-5)	Mouse	1:50	sc-25346, Santa Cruz Biotechnology, Dallas, TX, USA
	Dab1 (phospho-Y232)	Rabbit	1:100	ab78200; Abcam, Cambridge, UK
Secondary	Alexa Fluor^®^ 488 Affini-Pure Donkey Anti-Rabbit IgG (H + L) 711-545-152	Donkey	1:300	Jackson Immuno Research Laboratories, Inc., Baltimore, PA, USA
	Alexa Fluor^®^ 488 Affini-Pure Donkey Anti-Sheep IgG (H + L) 713-545-003	Donkey	1:300	Jackson Immuno Research Laboratories, Inc., Baltimore, PA, USA
	Alexa Fluor^®^ 488 Affini-Pure Donkey Anti-Mouse IgG (H + L) 711-545-154	Donkey	1:300	Jackson Immuno Research Laboratories, Inc., Baltimore, PA, USA

## Data Availability

The data assessed and reported here can be obtained from the authors upon reasonable request following ethical and privacy principles.
